# Occipital pressure injury in the beach-chair position: a report of two cases and a review of the literature

**DOI:** 10.1007/s00402-026-06560-5

**Published:** 2026-11-14

**Authors:** Bushra Alshanqiti, Mohamed Imam, Kishan Gokaraju, Abhinav Gulihar

**Affiliations:** 1 King Abdullah Medical Complex, Jeddah, Saudi Arabia; 2 King’s College Hospital, London, UK; 3 Ashford and St Peter’s Hospitals NHS Foundation Trust, Chertsey, UK

**Keywords:** Postoperative pressure alopecia, Superficial cervical plexus, Lesser occipital nerve, Beach-chair position, Shoulder arthroscopy

## Abstract

**Abstract:**

Pressure-related occipital complications of the beach-chair position have been attributed to headrest compression, prolonged immobilisation or systemic hypoperfusion. We present two patients who developed postoperative pressure alopecia and lesser occipital neuropraxia after shoulder arthroscopy on a T-MAX (Smith & Nephew, Andover, MA) beach-chair, used in its standard articulating-cradle and foam-mask configuration. Both remained normotensive throughout, with continuous cerebral oximetry showing no desaturation, and both were positioned in neutral alignment without rotation. One developed a well-demarcated patch of occipital alopecia after 92 min; the other developed lesser occipital neuropraxia after a 180-minute procedure. Both recovered fully, at four and five months respectively. Building on earlier reports implicating the retention straps alongside rotation and duration, we propose that in the near-upright position used here, at a trunk inclination of approximately 80 degrees, the load pressing on the occiput is supplied in part by the straps rather than by gravity alone. Because interface pressures were not measured, this remains a hypothesis requiring direct testing. We describe the layered padding and repositioning protocol subsequently adopted in our unit, emphasising pressure redistribution and repeated intraoperative checks of head position.

**Level of evidence:**

Level V, Case Report.

## Introduction

Arthroscopic surgery has become the preferred technique for many shoulder conditions, with the beach-chair position often used because it offers improved surgical access and visualisation of the glenohumeral joint [1]. The anatomical orientation of this position resembles that of an upright patient, which many surgeons find intuitive. Complications related to the position are nonetheless well documented and include cerebral hypoperfusion, deep vein thrombosis and airway compromise [2, 3].

Prolonged occipital pressure can also injure the superficial sensory nerves of the occiput and produce ischaemic injury to scalp hair follicles, presenting as postoperative (pressure) alopecia [2, 4].

These complications are uncommon but have been reported after shoulder surgery in the beach-chair position [2, 5, 6]. Reports involving rectangular and horseshoe-shaped headrests have implicated local compression, head and neck positioning, operative duration and the methods used to secure the head [2, 5]. These observations suggest that headrest geometry should be considered alongside positioning and other sources of focal loading.

We report two consecutive patients who developed pressure-related occipital complications after shoulder arthroscopy: alopecia in one and lesser occipital nerve neuropraxia in the other; both were performed on a T-MAX (Smith & Nephew, Andover, MA) table attachment with its integrated head holder, used in its standard configuration with the manufacturer-supplied sponge face mask and forehead and chin straps.

Our aim is to examine the potential contribution of face-mask and retention-strap tension to occipital loading in the near-upright position and to discuss practical precautions in the context of the existing literature. This report and literature review were prepared in accordance with the CARE (CAse REport) guidelines.

## Case reports

Both patients were positioned on a T-MAX beach-chair with the trunk inclined to approximately 80 degrees and the hips and knees flexed to 30 degrees. The T-MAX head support is a rigid articulating shallow-padded occipital cradle mounted to the chair on dual ball-and-socket joints, which are aligned to the patient’s head position and then clamped to hold that position for the duration of the procedure. In both cases the head was placed in the cradle in neutral cervical alignment, without axial rotation or lateral flexion, and was retained against the headrest by the manufacturer-supplied sponge face mask, buckled across the forehead and chin and tightened evenly in accordance with routine institutional practice. Strap tension was not measured or otherwise recorded.

No supplementary occipital padding, additional sponge-foam layer, foam roll, horseshoe-shaped headrest, rigid pin fixation or adhesive head tape was applied; this reflected our routine practice at the time. The hair was left loose in both patients, without braids, coils or elastic bands. Continuous non-invasive blood pressure, regional cerebral oxygenation (rSO₂) and processed-EEG depth-of-anaesthesia monitoring (SedLine) were performed in both cases using the Masimo platform (Masimo Corporation, Irvine, CA).

### Case 1

A 53-year-old man (body mass index 27 kg/m², American Society of Anesthesiologists physical status II, no diabetes, no previous scalp disease, normal hair density) underwent right shoulder arthroscopic rotator cuff repair under general anaesthesia with a regional interscalene block. Positioning followed the routine described above. Total operative time was 92 min. Mean arterial pressure was 71 mmHg throughout, with no hypotensive episode, defined as a mean arterial pressure below 60 mmHg, and stable cerebral oximetry.

At the first postoperative review the patient reported a focal area of hair loss in the left occipital region. Examination demonstrated a well-demarcated patch of complete alopecia measuring approximately 5 × 3 cm, without erythema, induration or signs of infection. A clinical diagnosis of postoperative pressure alopecia was made; biopsy and systemic therapy were not considered necessary and the patient was reassured. Hair regrowth began at two months and was complete by four months, with no residual scarring.

### Case 2

A 24-year-old man (body mass index 24 kg/m², American Society of Anesthesiologists physical status I, otherwise healthy) underwent left shoulder arthroscopic anterior and posterior labral repair with remplissage for recurrent anterior instability, in the presence of an engaging Hill-Sachs lesion. The labral tear was extensive and close to circumferential, and eight suture anchors were required. Surgery was performed under general anaesthesia with a regional interscalene block. Positioning again followed the routine described above. Total operative time was 180 min, at the upper end of the expected duration for this combined procedure. Mean arterial pressure was 71 mmHg throughout, with no hypotensive episode and stable cerebral oximetry.

Postoperatively the patient reported persistent numbness and dysaesthesia at the back of the head. Examination showed reduced sensation over the lateral occiput and mastoid region, corresponding to the cutaneous distribution of the lesser occipital nerve, without motor deficit. Symptoms had not improved at three months. The patient was anxious and had undertaken extensive online research, and a brain MRI obtained at his request was unremarkable. A clinical diagnosis of compressive lesser occipital neuropraxia was made. With reassurance and observation the symptoms resolved gradually over five months (Table 1).

**Table 1:** comparative perioperative characteristics, clinical presentation, clinical course, and outcomes of the two cases

Time point	Case 1 (53 M, rotator cuff repair)	Case 2 (24 M, labral repair with remplissage)
Surgery	Beach-chair, T-MAX cradle, no supplementary occipital padding; 92 min; MAP 71 mmHg throughout	Beach-chair, T-MAX cradle, no supplementary occipital padding; 180 min; MAP 71 mmHg throughout
First postoperative review	Focal left occipital hair loss, 5 × 3 cm, well demarcated	Persistent numbness and dysaesthesia over the lateral occiput
Examination	Complete alopecia patch; no erythema, induration or infection	Reduced sensation over lateral occiput and mastoid region; no motor deficit
Diagnosis	Postoperative pressure alopecia (clinical)	Compressive lesser occipital neuropraxia (clinical)
Investigations	None required	Brain MRI at patient request; unremarkable
3 months	Regrowth under way, having begun at 2 months	Symptoms unchanged
Resolution	Complete regrowth by 4 months; no scarring	Gradual full resolution by 5 months

## Discussion

These two cases illustrate two distinct but mechanistically related pressure complications of the beach-chair position: postoperative occipital alopecia and lesser occipital nerve neuropraxia. Both patients recovered fully, although the complications caused considerable anxiety. Neither case was associated with documented intraoperative hypotension, and in neither was the head rotated or laterally flexed. These observations direct attention to local occipital loading, while leaving the relative contributions of headrest contact, retention straps and other positioning factors unresolved.

The better characterised of the two conditions is pressure alopecia, a recognised but under-reported complication of prolonged surgical immobilisation, first described by Abel and Lewis in 1960 [7] and subsequently reported after cardiac, gynaecological, abdominal and maxillofacial procedures [4, 5]. The accepted mechanism is sustained external occipital pressure exceeding capillary perfusion pressure, approximately 32 mmHg, producing focal scalp ischaemia and hair-follicle injury [8, 9]. Steinmetz and Langemo measured occipital interface pressures during cardiac surgery, found values exceeding capillary perfusion pressure, and recommended scheduled head repositioning [10].

Reports outside orthopaedics describe pressure alopecia after prolonged procedures with limited head repositioning, including cases without documented intraoperative hypotension [8, 11]. These include supine surgery, cardiac, abdominal, gynaecological and prolonged microsurgical reconstruction, in which the head rests on a horseshoe, head ring or gel donut and gravity acting vertically on the occiput is the dominant source of contact pressure. Reddy et al. reported alopecia after open fixation of a proximal humeral fracture in the beach-chair position, using a horseshoe-shaped headrest during an operation lasting three hours and 25 min [5]. Our first case differed in headrest configuration and operative duration: a rigid articulating T-MAX headrest with a shallow padded occipital cradle was used, and the operation lasted 92 min. Its occurrence highlights the need to assess local contact and pressure distribution even when a padded cradle is used.

Our second case represents a different type of injury, and the distinction matters when considering risk. Pressure alopecia and lesser occipital neuropraxia share focal occipital compression as their proximate cause, but their pathophysiology, and therefore their risk profile, diverges. Pressure alopecia is an ischaemic injury of the hair follicle. It arises when sustained interface pressure at the occiput exceeds scalp capillary perfusion pressure, producing follicular hypoxia, and the depth and reversibility of the lesion follow a recognised pressure-time dose response in which both the magnitude of contact pressure and the duration of loading matter [12]. Duration is therefore an important variable, which is why pressure alopecia is reported predominantly after prolonged supine procedures in which the occiput bears the full vertical weight of the head [8, 10, 11].

Lesser occipital neuropraxia, by contrast, is a focal compression neuropathy of an anatomically superficial nerve. The lesser occipital and greater auricular branches of the superficial cervical plexus pierce the investing cervical fascia at the posterior border of sternocleidomastoid and then run subcutaneously over the mastoid and lateral occiput, lying immediately beneath the skin with no intervening muscle or fat to distribute load [13, 14]. At that location even a brief episode of focal compression between the headrest and the patient’s own skull can produce segmental demyelination or, with greater force, axonal injury [13]. Two observations in the literature illustrate the consequence. First, Tahir and Corbett described permanent lesser occipital neurotmesis following shoulder arthroscopy on a horseshoe-shaped headrest after an operative time of approximately 30 min [6], suggesting that focal compression over a vulnerable nerve can be significant even during short procedures. Second, lesser occipital nerve injury has been reported across a wide range of headrest designs: the rectangular headrest [2], the horseshoe headrest [6, 15, 16], the standard universal headrest [17], and the intubation pad-type headrest [18]. These reports suggest that injury is not confined to a single headrest geometry and that the anatomical vulnerability of these superficial nerves warrants consideration alongside headrest contact and head positioning.

Our second case adds the shallow-padded articulating cradle to these reports of positioning-related nerve injury. Pressure alopecia after supine surgery is well established [4, 7, 8, 11], and injuries to superficial cervical plexus branches in the beach-chair position has been reported repeatedly since 2005 [2, 6, 15–18]. To our knowledge, neither pressure alopecia nor lesser occipital neuropraxia has previously been reported with the T-MAX articulating cradle. The alopecia case is additionally unusual for the short operative time of 92 min. Retention straps have also been implicated previously. Park and Kim, reporting cervical plexus neuropraxia on a rectangular headrest in 2005, attributed injury severity to the degree of head and neck rotation and deviation, the duration of the procedure, and compression by the head strap and elastic bandage used to fix the head to the headrest [2]. Our contribution is a mechanical hypothesis describing how strap tension, head weight and forces transmitted through the neck combine to load the occiput in the near-upright position.

**Fig. 1:**
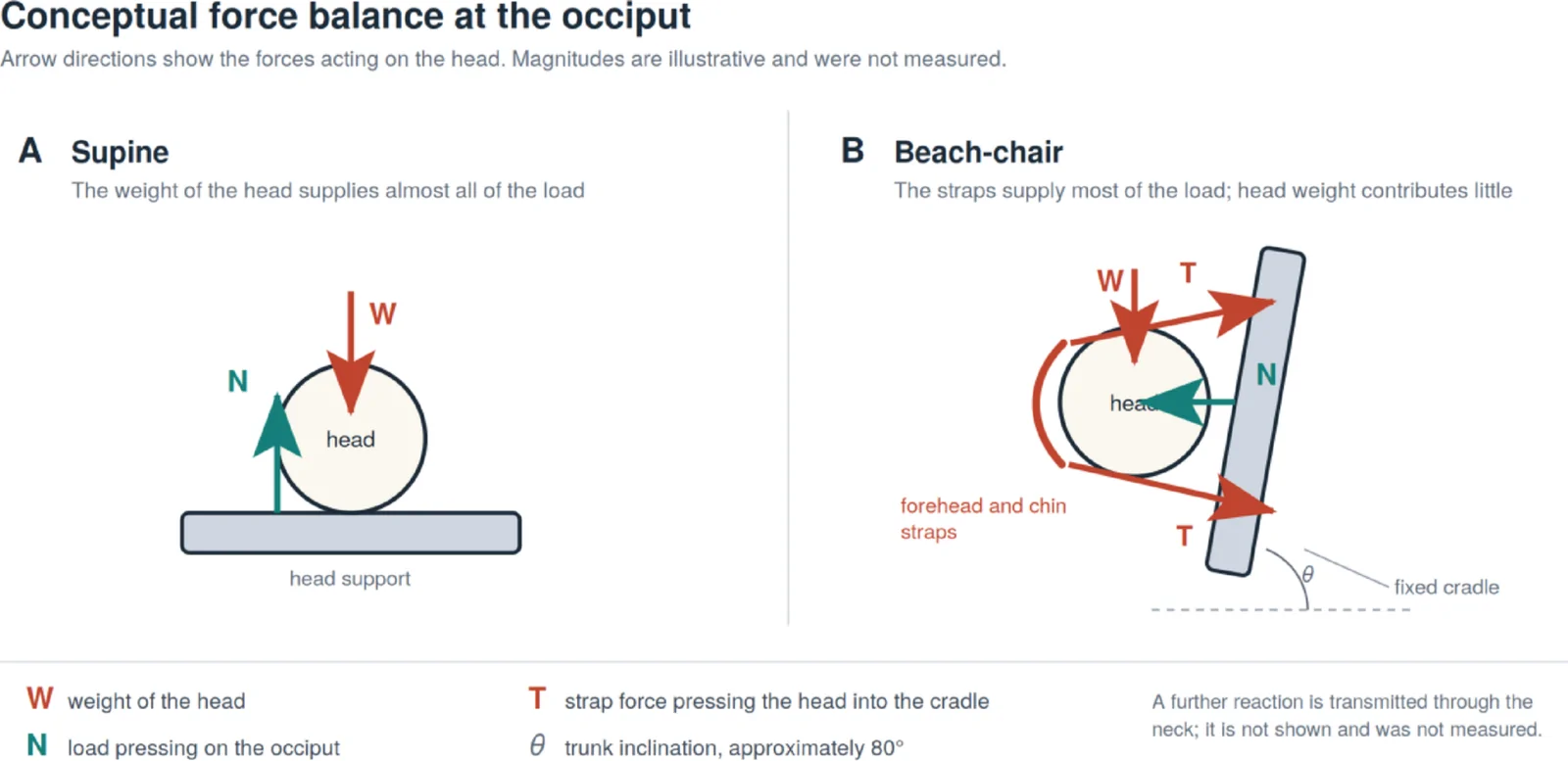
Conceptual force balance at the occiput. (**A**) Supine position: the weight of the head (W) acts vertically and supplies almost all of the load pressing on the occiput, so the support reaction (N) is close to head weight. (**B**) Beach-chair position: the head is held against a fixed cradle by the forehead and chin straps. At a trunk inclination of approximately 80 degrees, the proposed model considers contributions to occipital loading from strap force (T), the perpendicular component of head weight and a reaction transmitted through the neck. W, weight of the head; N, load pressing on the occiput; T, the component of forehead and chin strap force that presses the head into the cradle; θ, trunk inclination. The neck reaction is not shown and was not measured. Arrow directions show the forces acting on the head; magnitudes are illustrative and are not derived from measured interface pressures

This hypothesis rests on the mechanics of the position, which differ from those of supine surgery. In supine surgery, the head rests on the headrest under its own weight, so gravity drives the occiput vertically into the support and is the principal source of contact pressure, with the normal load approximating head weight. In the beach-chair position at a trunk inclination of 70 to 80 degrees, most of the head’s weight is transmitted along the line of the cervical spine rather than perpendicular to the cradle. The normal load at the occiput is represented as the sum of three terms: the components of forehead and chin strap tension acting perpendicular to the cradle, the perpendicular component of head weight, and a reaction transmitted through the neck. This comparison is set out in Fig. 1.

The relative size of these terms is central to the proposed explanation. At a trunk inclination of approximately 80 degrees, the perpendicular component of head weight falls to roughly 0.17 W in this model, while the neck-reaction term is unquantified and the strap term is set entirely by how firmly the face mask is tightened. The two cases are consistent with this explanation: injury occurred on a padded cradle, without documented hypotension or head rotation, and in one instance after only 92 min. Interface pressures and strap tension were not measured, so these cases cannot establish which loading component predominated. Quantifying the occiput–cradle interface pressure under both supine and beach-chair loading conditions, across a range of strap tensions, would test the proposed relationship between strap tension, position and contact pressure. Its clinical relevance does not depend on the exact proportion of load attributable to strap tension. Headrest geometry is determined by the equipment available in a given theatre, whereas strap tension, head alignment within the cradle and the frequency of repositioning can be adjusted by the operating team, at no cost, on any equipment.

This mechanical hypothesis should be considered within the multifactorial risk profile of these injuries. Focal occipital compression offers a possible shared mechanism for pressure alopecia and lesser occipital neuropraxia, while neuropraxia may also arise from head and neck positioning [19]. Strap tension is one potential source of local loading, alongside headrest contact and alignment. Patient and procedural factors influence whether a given load produces injury, the tissues affected and the severity of the resulting lesion.

Operative duration remains relevant, nerve injury can occur after short procedures [6], while longer operations, such as the 180-minute procedure in Case 2, entail more sustained contact. Contact area is another factor, since capillary perfusion pressure can be exceeded under modest loads where the contact area is small or the underlying support does not conform, particularly over the external occipital protuberance [8, 10]. Head rotation and asymmetric strap tension concentrate load onto a smaller area of scalp and stretch the superficial cervical plexus branches across the underlying bone, consistent with the positioning and fixation concerns raised by Park and Kim [2]. Headrest geometry also matters, in that bare rigid or horseshoe headrests concentrate load along narrow edges while a conforming padded cradle spreads contact and reduces focal pressure, although injury can still occur [2, 6, 15, 16]. Patient-level factors including hair density, scalp thickness, body habitus, microvascular disease and anatomical variation in the course of the cervical plexus branches modulate individual susceptibility [4, 8]. Systemic factors such as hypotension and impaired cerebral or scalp perfusion are recognised amplifiers, although neither was present in our patients [1, 8, 11].

## Prevention protocol

**Fig. 2:**
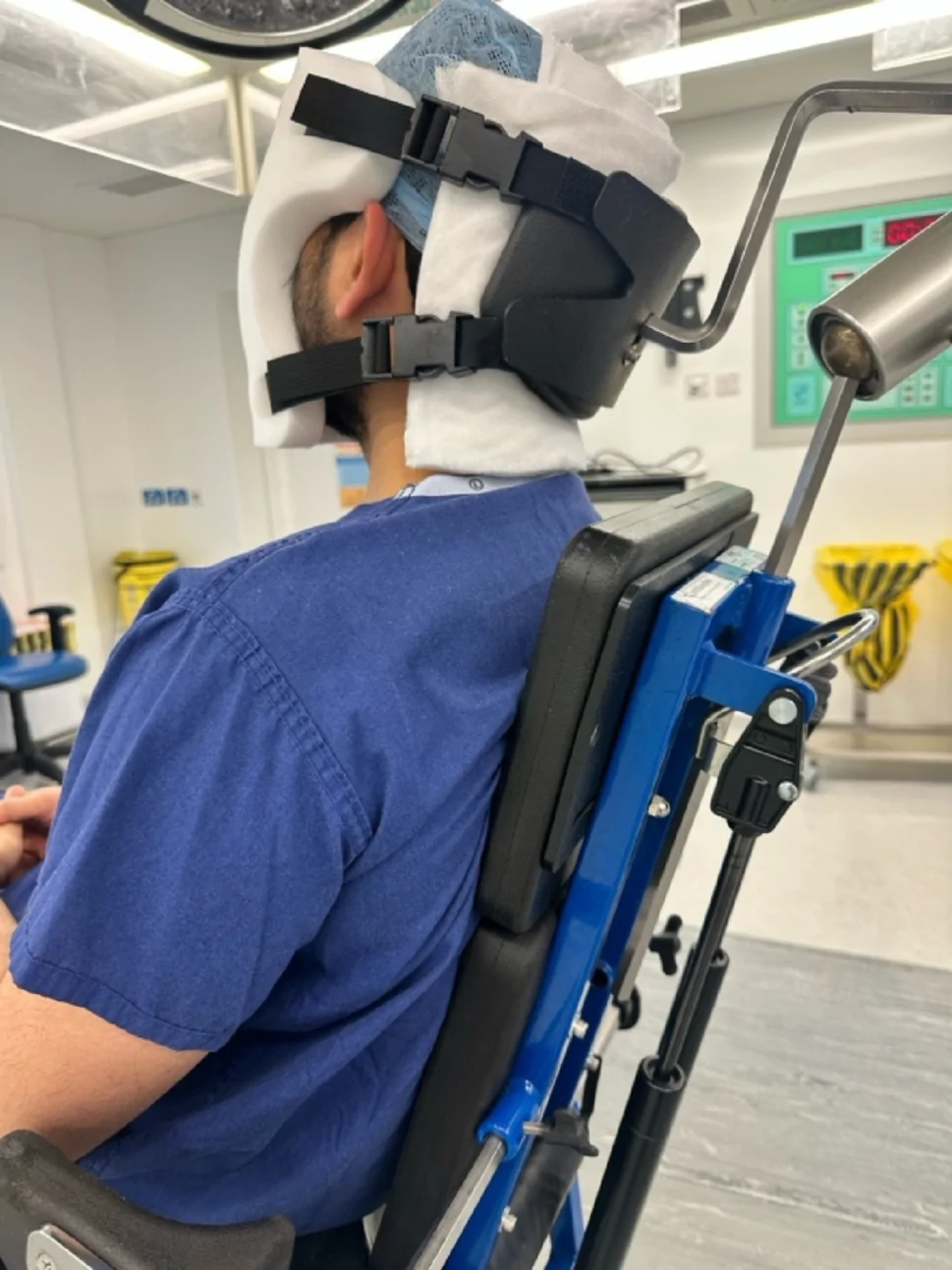
Posterior view of the beach-chair set-up illustrating the supplementary padding protocol adopted following the two index cases. The rigid articulating T-MAX headrest is shown with its shallow padded occipital cradle augmented by an overlying conforming sponge-foam pad shaped to spread contact across the whole occiput, together with a supplementary foam roll at the cervico-occipital junction. This configuration was not used during the index operations

**Fig. 3:**
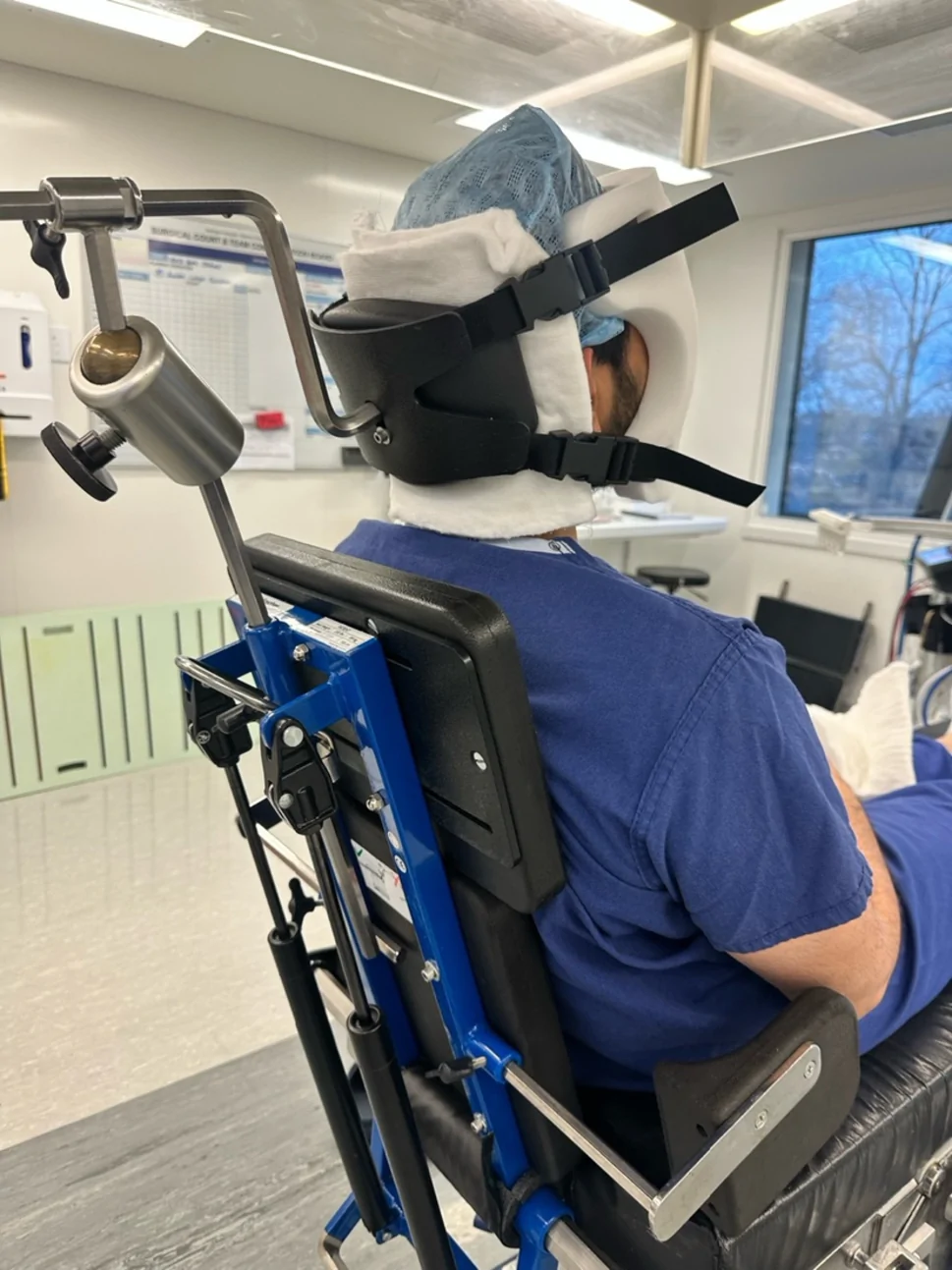
Lateral view of the same set-up, showing the additional padding supporting the head and neck against the rigid headrest. The padding is intended to spread occipital contact and reduce focal pressure over the external occipital protuberance. As in Fig. 2, this configuration represents post-incident practice and was not used in Cases 1 and 2

In direct response to these two cases, we adopted a layered supplementary padding protocol with scheduled head repositioning and repeated intraoperative verification of head position. This builds on established padding and positioning precautions for superficial cervical plexus injury [2, 16], with an emphasis on team awareness and prevention of positioning-related harm [20]. It is illustrated in Figs. 2 and 3 and is intended to reduce, though not to eliminate, the risk of pressure-related occipital injury.

The protocol retains the baseline positioning described above and adds four measures. First, a double layer of soft padding approximately 2 cm thick and 20 × 25 cm is placed over the standard T-MAX cradle, shaped to spread contact across the whole occiput rather than a focal point, together with a flat foam roll supporting the cervico-occipital junction to disperse load away from the external occipital protuberance. Second, the manufacturer-supplied padded face mask is applied with one finger-breadth of slack at the forehead and chin straps, sufficient to prevent anterior displacement of the head without indenting the skin. Third, the anaesthetic team rechecks head alignment after draping and at 30-minute intervals, and a brief lift-and-replace repositioning of the head is performed whenever surgical exposure allows, particularly once operative time exceeds 120 min; for combined or revision procedures with an anticipated duration beyond 150 min, scheduled repositioning and reinforced consent are adopted from the outset. Fourth, the occiput is examined in recovery and at the first follow-up visit, and the patient is asked specifically about scalp tenderness, sensory disturbance and hair loss, since early recognition and reassurance avoid unnecessary investigation. Throughout, mean arterial pressure is maintained within a patient-appropriate range under continuous cerebral oximetry and any hypotension is corrected promptly, recognising that normotension alone does not remove the risk.

The consent conversation deserves equal emphasis. Both patients were distressed by their symptoms, and one underwent an MRI at his request. We now explicitly discuss the possibility of postoperative pressure alopecia and occipital sensory disturbance during preoperative consent for beach-chair surgery.

.

## Limitations

This is a two-patient case series and cannot establish incidence or causation. Intraoperative occiput-cradle interface pressures were not measured, and strap tension was not quantified or recorded, so the force-balance account proposed here remains a hypothesis and the cases are consistent with, rather than demonstrative of, the mechanism. We cannot separate the contribution of strap tension from those of contact area, cradle conformity and individual anatomy or exclude other positioning-related causes of neuropraxia. Direct quantification under supine and beach-chair loading, and across a range of padding and strap-tension configurations, would test the hypothesis and is the subject of ongoing work in our unit. Both diagnoses were clinical: neither nerve conduction studies nor scalp biopsy was performed, although both patients recovered fully, which is consistent with neuropraxia and with non-scarring pressure alopecia respectively. The prevention protocol is pragmatic, derived from case-level evidence and the published literature, and has not been evaluated prospectively; we cannot state what proportion of risk it removes.

## Conclusion

Pressure-induced occipital alopecia and lesser occipital neuropraxia are uncommon but clinically important complications of shoulder arthroscopy in the beach-chair position. Both occurred here in normotensive patients under continuous cerebral oximetry, positioned without head rotation on a standard T-MAX articulating headrest with a shallow padded occipital cradle and no supplementary occipital padding. The alopecia followed a 92-minute procedure. We propose that, at a trunk inclination of 70 to 80 degrees, retention-strap and face-mask tension may contribute to occipital loading alongside head weight and forces transmitted through the neck. Direct measurements are needed to establish their relative contributions. Operative duration, contact area, headrest geometry, head and neck positioning, patient anatomy and systemic perfusion remain important considerations. Our subsequent protocol builds on established precautions, emphasising layered supplementary padding, controlled strap tension, repeated intraoperative verification of head position, scheduled repositioning, haemodynamic vigilance, postoperative occipital examination and explicit preoperative consent. These measures are intended to minimise the risk of pressure-related occipital injury.

## Patient consent statement

## Data Availability

No datasets were generated or analysed during the current study.

## Declarations

### Reporting guideline

This case series was prepared in accordance with the CARE (CAse REport) guidelines.
